# Further investigation of the psychometric properties of the insulin treatment appraisal scale among insulin-using and non-insulin-using adults with type 2 diabetes: results from diabetes MILES – Australia

**DOI:** 10.1186/1477-7525-12-87

**Published:** 2014-06-06

**Authors:** Elizabeth Holmes-Truscott, Frans Pouwer, Jane Speight

**Affiliations:** 1 The Australian Centre for Behavioural Research in Diabetes, Diabetes Australia, Vic, 570 Elizabeth Street, Melbourne 3000, VIC, Australia; 2 School of Psychology, Deakin University, 221 Burwood Highway, Burwood 3125, VIC, Australia; 3 Department of Medical and Clinical Psychology, Centre of Research on Psychology in Somatic diseases (CoRPS), Tilburg University, Postbus 90153, 5000 LE, Tilburg, The Netherlands; 4 Applied Health Psychology Research Ltd, 16 Walden Way, Hornchurch RM11 2LB, United Kingdom

**Keywords:** Psychological insulin resistance, Type 2 diabetes, Questionnaire, Psychometric validation

## Abstract

**Background:**

Negative attitudes towards insulin are commonly reported by people with type 2 diabetes mellitus (T2DM) and can act as a barrier to timely insulin initiation. The Insulin Treatment Appraisal Scale (ITAS) is a widely used 20-item measure of attitudes towards insulin. While designed for completion by both insulin using and non-insulin using adults with T2DM, its psychometric properties have not been investigated separately for these groups. Furthermore, the total score is routinely reported in preference to the published two-factor structure (negative/positive appraisals). Further psychometric validation of the ITAS is required to examine its properties.

**Methods:**

The ITAS was completed by a subgroup of 748 Diabetes MILES – Australia study participants with T2DM, who were either insulin using (n = 249; 45% women; mean age = 58 ± 9 years; mean diabetes duration = 13, SD = 8 years) or non-insulin using (n = 499; 47% women; mean age 57 ± 9 years; mean diabetes duration 7 ± 6 years). We replicated the psychometric analyses reported in the ITAS development paper. In addition, we explored factor structure and investigated internal consistency separately for the insulin using and non-insulin using samples.

**Results:**

Factor analyses supported a two-factor structure with good internal consistency (negative subscale α = .90; positive subscale α = .69). Scale performance differed slightly in the insulin using and non-insulin using samples, with some items loading inconsistently between groups. A one-factor solution was not supported in either sample, with the positive items and some negative items failing to load adequately. Consistent with prior research, negative appraisals were significantly more common among non-insulin using participants compared to those using insulin (*d* = 1.04), while the positive subscale score did not discriminate between groups.

**Conclusions:**

The data supported a two factor structure and the positive subscale did not discriminate between insulin using and non-insulin using participants. As such, we recommend use of the negative subscale score in preference to the ITAS total score, and suggest close attention is paid to the relevance of the positive items in the given population.

## Background

Type 2 diabetes mellitus (T2DM) is a progressive condition and most people with this condition will eventually require exogenous insulin to maintain haemoglobin A1c within recommended targets [1,2]. However, a quarter of adults with T2DM report being unwilling to begin insulin therapy [3], commonly reporting concerns about the necessity of insulin, as well as the physical, social and symbolic adverse consequences of insulin use [4]. These negative attitudes, known as ‘psychological insulin resistance’ (PIR), may lead to delays in insulin initiation or sub-optimal use once insulin is prescribed [3,5-8]. PIR is a complex construct that does not simply equate to “fear of the needle”, as people can base their reluctance to use insulin on many different aspects of the therapy. The construct has been operationalised through assessment of attitudes toward insulin (insulin appraisal) [3,9-13].

Two scales have been developed and validated specifically to measure attitudes towards insulin held by people with T2DM. The 14-item ‘Barriers to Insulin Treatment’ (BIT) self-report questionnaire measures attitudes towards insulin amongst people with non-insulin-treated T2DM [13]. BIT items commonly refer to the physical aspects of insulin use or technical concerns (e.g. side effects, pain) rather than the symbolic meaning of insulin initiation (e.g. feelings of failure/self-blame or increased diabetes severity). The Insulin Treatment Appraisal Scale (ITAS) is a 20-item questionnaire, including 16 statements referring to barriers to insulin use and four referring to its benefits (12). Unlike the BIT, the more commonly used ITAS was developed and validated for use by people with T2DM regardless of current treatment type, with the advantage of enabling assessment both before and after insulin initiation [12]. For non-insulin using respondents, the ITAS assesses expectations about future insulin use, while for those already using insulin, the measure is used to evaluate actual experience with insulin use.

The clinical relevance of the ITAS has been demonstrated. In cross-sectional studies, a difference has been observed between insulin using and non-insulin participants in total ITAS scores of approximately one standard deviation [12,14]. Longitudinal research indicates that the ITAS is sensitive to treatment change from oral medication to insulin injections [15]. Furthermore, higher ITAS scores (indicating more negative appraisal of insulin) are associated with being hypothetically less ‘willing’ to begin insulin if recommended [10]. Previous research has identified associations between ITAS scores and general and diabetes-specific emotional wellbeing among people with T2DM [12,16].

Initial investigation of the structure of the ITAS revealed it to have a two-factor solution, identifying a positive subscale (benefits) and a negative subscale (barriers) [12]. Despite reporting low item commonalities in a one-factor structure, scale developers proposed the use of an ITAS total score (summation of all 20 items), to indicate a person’s overall insulin appraisal. Since the original US study [12], no further work has been published regarding the validation of the two-factor or one-factor structure, and the ITAS total score has been used most commonly [9,10,16]. Despite reports that the ITAS is psychometrically sound for both insulin using and non-insulin using participants [12], psychometric analyses have not been reported separately for these groups. Given that these groups may have either quantitatively or qualitatively different attitudes towards insulin, based on expectations or actual experience, an investigation is needed of how the scale performs psychometrically in each of these groups separately.

Further psychometric analyses are required to assess the appropriateness of the scale in the two separate samples for which it was intended, as well as to further evaluate the validity of the ITAS total score. Thus, our aim was to further examine the psychometric properties of the ITAS separately among insulin using and non-insulin using adults with T2DM in Australia.

## Methods

This analysis utilises a subset of data from Diabetes MILES – Australia, a large-scale, national, cross-sectional survey of Australian adults (aged 18 to 70 years) diagnosed with either type 1 diabetes or T2DM. The survey was conducted in July – August 2011. A detailed description of the methods, response rates and questionnaires has been published elsewhere [17]. Diabetes MILES – Australia received ethics approval from the Deakin University Human Research Ethics committee (reference number: 2011–046).

### Participants

Diabetes MILES – Australia surveys were sent out to a random sample of 15,000 National Diabetes Services Scheme (NDSS) registrants. The NDSS register includes >1.1 million registrants living with diabetes in Australia, of whom 87% have T2DM (http://www.ndss.com.au/en/Research/Data-Snapshots/). Survey booklets were matched to the recorded diabetes diagnosis and treatment (confirmed at registration by a health professional): T1DM, T2DM Insulin using or T2DM non-insulin.

The survey was also made available online nationally, with respondents required to self-report their type of diabetes in order to receive the appropriate survey version. The database was cleaned to validate survey versions against self-report diabetes diagnosis, age at diagnosis, and treatment type to ensure the highest level of accuracy possible given the self-report nature of the survey.

Overall, there were 3,338 eligible respondents, of whom 1,962 reported living with T2DM. Of these, 49% (n = 953) were women, the mean ± SD age was 59 ± 9 years and 37% (n = 724) were using insulin. The current analysis focuses on a subsample of participants with T2DM who were invited to complete the ITAS [12] and reported their diabetes treatment type as either insulin using, requiring oral anti-hyperglycaemic tablets or following lifestyle recommendations. Participants reporting use of glucagon-like peptide-1 (GLP-1) agonist treatment were excluded from the current study. Like insulin, GLP-1 agonist is administered via injection, but differs from insulin use in a number of ways including, its efficacy; potential side effects (e.g. weight loss versus possible weight gain when using insulin) [18]; and the associated stigma of the treatment [19-22]. Therefore participants using GLP-1 injections are not easily classified within either treatment group (insulin using and non-insulin using) relevant to this study.

### Measures

The Diabetes MILES – Australia surveys included a set of core measures (completed by all respondents) and various additional measures (included in one or more of the six survey versions). Full details of the measures are published elsewhere [17]. Variables of interest to this study include: demographics (age, gender, relationship status, whether employed in paid work, education level, and body mass index), diabetes health status (self-reported diabetes type, primary treatment and years living with diabetes), and the ITAS [12].

The ITAS asks respondents to indicate their level of agreement (‘strongly disagree’ = 1 to ‘strongly agree’ = 5) with 20 statements. Scores for 16 negatively-worded items are summed to provide a ‘negative appraisal’ score (16–80); scores for four positively-worded items are summed to provide a ‘positive appraisal’ score (4–20); all twenty items are summed (with positively-worded items reversed) to form a ‘total’ score (20–100). Higher ‘total’ and ‘negative appraisal’ scores indicate more negative attitudes, while higher ‘positive appraisal’ scores indicate more positive attitudes towards insulin. Permission to use the ITAS was granted by the copyright holders.

### **Stat**i**stical analysis**

Statistical analysis was undertaken using SPSS version 21 (Chicago, USA). Frequencies, means and standard deviation were obtained for ITAS and relevant demographics for insulin using and non-insulin using participants. Acceptability of the scale was assessed by examining completion rates and identifying ceiling effects for negative ITAS items and floor effects for positive ITAS items (i.e. >20% scoring minimum/maximum response) [23]. To replicate the methods described in the ITAS development paper [12], we conducted exploratory factor analysis (EFA) with oblimin rotation on the 20-item scale for the whole sample, as we expected the factors to be correlated. When inspecting the Eigenvalues, we used the Kaiser-criterion (Eigenvalue >1) and reviewed the scree plot to determine the maximum number of factors. In accordance with the original development paper, item loadings were considered optimal if they were >0.40 on one factor and <0.30 on any other factor [12]; a less conservative criterion of loading >0.30 (without concern for double loadings) was also adopted [24]. To replicate the hypothesised optimal scale structure, and to assess the suitability of the total score, forced two-factor and one-factor solutions were conducted respectively. Internal consistency was estimated using Cronbach’s alpha and Guttmans λ_2_[25], where ≥0.70 and <0.90 was considered reasonable. Item-total correlations were also calculated, with a score of <0.20 taken to indicate a poor relationship with the total scale score. These psychometric analyses were conducted for the insulin-using and non-insulin using samples separately and a forced one-factor solution was also investigated.

Known-groups validity was explored by comparing mean ITAS total, negative and positive scores between treatment groups. Student t-tests or chi-squares were conducted to assess between-groups differences in ITAS scores. The association between demographics and total ITAS scores between groups was explored using Student t-tests and bivariate correlations. Statistical tests are two-sided with differences accepted at a significant level of *p* < 0.05. Effect sizes are reported using Cohen’s *d*.

## Results

Overall, 887 participants with T2DM completed the Diabetes MILES – Australia survey versions in which the ITAS was included. Of these, 24 participants were excluded due to unreported treatment type or reported use of GLP-1 agonist injections. A further 115 (12.9%) participants were excluded due to non-completion of at least one ITAS item (see ‘Acceptability’ for further details). Of the 748 eligible respondents, one third were using insulin to manage their diabetes. Participant characteristics are shown in Table 1. Within the current sample, 193 participants (26%) completed the online version of the survey, and 555 (74.2%) completed the hardcopy survey.

**Table 1:** Self-reported demographics and clinical characteristics of insulin using and non-insulin using participants

	**Non-insulin users**	**Insulin users**
N (%)	499 (67%)	249 (33%)
Female sex	233 (47%)	112 (45%)
Age - years	57 ± 9 (22 – 70)	58 ± 9 (21 – 70)
Employment - in paid work	279 (56%)	104 (42%)
*Education*		
Low	32 (7%)	36 (16%)
Medium	314(66%)	157 (67%)
High	132 (28%)	40 (17%)
Having a partner	369 (75%)	178 (73%)
Diabetes duration - years	7 ± 6 (<1 – 35)	13 ± 8 (<1 – 42)
*Primary Treatment*		
Lifestyle modifications	130 (26%)	-
Blood glucose lowering tablets	369 (74%)	-
Insulin injections	-	247 (99%)
Insulin pump	-	2 (1%)
BMI	31 ± 7 (13 – 78)	34 ± 9 (15 – 92)

NB. Valid percentage reported as n values vary due to missing data on individual variables.

Data are mean ± standard deviation or N (%).

### Acceptability

Non-insulin using participants were slightly more likely to have missing data; 83 (14%) non-insulin participants compared to 32 (11%) insulin-using participants missed at least one item. Of the non-insulin using participants with missing data, almost half (n = 39) skipped all 20 items. Thirty-three missed just one item and the remaining 11 missed between 2 and 19 items. The majority of insulin using participants with missing data skipped only 1 item (n = 26), and a further six skipped between 2 and 15 items: none skipped the entire scale. Excluding those who missed the whole scale, each of the 20 ITAS items was completed by ≥98.2% of non-insulin using participants and, similarly, by ≥98.2% of insulin using participants.

Among the four positively-worded ITAS items, no floor effects were apparent amongst either insulin or non-insulin using participants (i.e. ≤20% of participants strongly disagreed with the benefits of insulin). Ceiling effects were apparent for four of the 16 negatively worded items among non-insulin using participants (>20% and <32% strongly agreed with items 1, 2, 5 and 6) but none were apparent among insulin using participants.

### Scale structure: whole sample

EFA analyses conducted on the whole sample revealed a maximum of four factors with an Eigenvalue >1, explaining 57.1% of the total variance. The Eigenvalue for (and variance explained by) each factor respectively was 6.7 (33.4%), 2.3 (11.7%), 1.3 (6.4%), and 1.1 (5.7%). Factors three and four were most easily interpretable: the four ‘benefits’all loaded onto one factor suggesting a ‘positive appraisal’ subscale, and the two side-effect items (‘increases risk of hypoglycaemia’ ‘weight gain’) loaded onto another factor. Eleven negative appraisal items loaded onto the first factor with the remaining three loading onto the second factor, with no clear interpretation for either.

Given the minimal additional variance explained by a 3- or 4-factor solution, and the aim to replicate the scale structure previously reported [12], a three-factor solution was not investigated. The two-factor solution explained 45% of the total variance with the first factor including all negatively-worded items except ‘weight gain’ and the second factor including the four positively-worded items. Only item 18 (‘family and friends concerns’) loaded >0.3 on more than one factor. The correlation between factors was low (r = 0.06), suggesting a Varimax rotation would be more suitable, but the results of this rotation did not differ from the loading pattern obtained using the oblique rotation. A forced one-factor solution explained 33.4% of the variance, with all four positive items and the ‘weight gain’ item failing to load sufficiently. Overall, reliability was satisfactory for the 20-item scale (α = 0.87; λ_2_ = 0.89), the 16-item negative subscale (α = 0.90; λ_2_ = 0.91), and for the positive subscale (α = 0.69; λ_2_ = 0.69). Table 2 displays both the forced one-factor and two-factor solutions, with item loadings for the whole sample (described here) and by treatment type (described below).

**Table 2:** Forced 1-factor and 2-factor EFA of the ITAS: whole sample and by treatment type

**Item**		**Whole sample**			**Non-insulin users**			**Insulin users**		
		**Total scale**	**Two factor**		**Total scale**	**Two factor**		**Total scale**	**Two factor**	
			**Negative subscale**	**Positive subscale**		**Negative subscale**	**Positive subscale**		**Negative subscale**	**Positive subscale**
**1**	Taking insulin means I have failed to manage my diabetes with diet and tablets	0.55	0.54		0.54	0.48		0.40	0.37	
**2**	Taking insulin means my diabetes has become much worse	0.60	0.59		0.59	0.48	-0.38	0.43	0.39	
**3^**	Taking insulin helps to prevent complications of diabetes			0.69			0.71			0.57
**4**	Taking insulin means other people see me as a sicker person	0.65	0.64		0.64	0.54	-0.36	0.59	0.59	
**5**	Taking insulin makes life less flexible	0.72	0.71		0.70	0.60	-0.33	0.64	0.69	
**6**	I'm afraid of injecting myself with a needle	0.65	0.66		0.58	0.60		0.52	0.51	
**7**	Taking insulin increases the risk of low blood glucose levels (hypoglycaemia)	0.44	0.43		0.37			0.47	0.47	
**8^**	Taking insulin helps to improve my health			0.76			0.72			0.81
**9**	Insulin causes weight gain				0.43	0.43		0.37	0.37	
**10**	Managing insulin injections takes a lot of time and energy	0.74	0.74		0.69	0.70		0.68	0.69	
**11**	Taking insulin means I have to give up activities I enjoy	0.72	0.73		0.70	0.74		0.67	0.69	
**12**	Taking insulin means my health will deteriorate	0.70	0.72		0.66	0.73		0.71	0.66	
**13**	Taking insulin is embarrassing	0.71	0.73		0.69	0.78		0.70	0.71	
**14**	Injecting insulin is painful	0.71	0.72		0.69	0.74		0.65	0.66	
**15**	It is difficult to inject the right amount of insulin correctly at the right time every day	0.68	0.69		0.67	0.71		0.51	0.50	
**16**	Taking insulin makes it more difficult to fulfil my responsibilities (at work, at home)	0.79	0.80		0.75	0.80		0.73	0.71	
**17^**	Taking insulin helps to maintain good control of my blood glucose			0.74			0.73			0.74
**18**	Being on insulin causes family and friends to be more concerned about me	0.59	0.56	-0.32	0.60	0.47	-0.42	0.40	0.48	
**19^**	Taking insulin helps to improve my energy levels			0.55			0.50			0.63
**20**	Taking insulin makes me more dependent on my doctor	0.62	0.61		0.64	0.58		0.48	0.51	
**Cronbach’s alpha**		**0.87**	**0.90**	**0.69**	**0.85**	**0.89**	**0.69**	**0.84**	**0.85**	**0.68**
**Total variance explained**		**33.4%**	**45%**		**32%**	**44.9%**		**27.0%**	**38%**	

NB. EFA conducted using oblimin rotation. Loadings < ±0.30 have been suppressed from the table for clarity of interpretation.

^positive ITAS items.

### Scale structure: by treatment type

In the non-insulin using sample, inspection of the Eigenvalues after EFA revealed a maximum of four factors, explaining 57.2% of the total variance. The Eigenvalues for (and variance explained by) each factor respectively was 6.4 (32.0%), 2.6 (12.9%), 1.4 (6.8%), and 1.1 (5.4%). Only factor four was interpretable, with all four ‘benefits’loading >0.4, suggesting a ‘positive appraisal’ subscale. Item 7 (‘increased risk of hypoglycaemia’) did not load >0.4 on any factor and six other items loaded ≥ ±0.3 onto more than one factor.

An EFA conducted for the insulin using participants revealed a maximum of five factors, explaining 57% of the total variance. The Eigenvalues for (and variance explained by) each factor respectively was 5.4 (27.0%), 2.2 (10.9%), 1.5 (7.7%), 1.2 (6.1%), and 1.0 (5.2%). Once more, one factor included satisfactory loadings for all four positive items while the other factors were unclear. Ten items loaded ≥ ±0.3 on two or more factors and two items did not load at all (‘weight gain’ and ‘concern from family and friends’).

The two-factor solution within the non-insulin using group explained 44.9% total variance, with the first factor including 15 of the 16 negative items, and the second factor including all four positive items. Item 7 (‘increases risk of hypoglycaemia’) did not load on either factor, or four of the negative items double-loaded (≥ ± 0.3) on both factors. Amongst the insulin using group, a two-factor solution explained 38% variance. Factor one included all 16 negative items and factor two included the four positive items.

A forced one-factor solution explained 32% and 27% of the total variance in the non-insulin using and insulin using samples respectively. The positive items did not load in either group. In the non-insulin using sample, all negative items except for item 7 (‘increases risk of hypoglycaemia’), loaded onto the factor and in the insulin using sample only item 9 (‘weight gain’) did not load.

In the non-insulin using sample, Cronbach’s alpha was 0.85 for the 20-item scale, 0.89 for the 16-item negative subscale, and 0.69 for the positive subscale. Guttmans λ_2_ was 0.87, 0.90, and 0.71 respectively. Internal consistency was similar for insulin using participants: for the total scale α = 0.84 and λ_2_ = 0.85, for the negative subscale α = 0.85 and λ_2_ = 0.87, and for the positive subscale α = 0.68 and λ_2_ = 0.69.

Within the total scale, all positive items displayed low item-total correlations; <0.1 for non-insulin participants and <0.32 for insulin using participants. All item-total correlations for negatively worded items were >0.2 for both non-insulin and insulin using participants. When exploring the negative and positive subscales separately, all item-total correlations were above the >0.2 cut off for non-insulin using participants (negative subscale range = 0.31-0.69, positive subscale range = 0.36-0.58) or for insulin using participants (negative subscale range = 0.35- 0.63, positive subscale range = 0.38-0.58).

### Known-groups validity

Table 3 displays, by treatment type, the mean and standard deviation for each of the ITAS items, total score, positive subscale and negative subscale, the *t*-test significance results and effect sizes showing between group differences, as well as the percentage who agreed or strongly agreed with each item. Non-insulin using participants reported significantly higher (more negative) scores compared to the insulin using participants on all negatively-worded items, except for ‘insulin causes weight gain’, for which insulin using participants reported higher (more negative) scores. Moderate effect sizes were found between groups for 9 of the 16 negative items (*d* range = 0.54-0.76), and large effect sizes for 4 items (*d* range = 0.86-1.08). Item 6 (‘I’m afraid of injecting myself with a needle’) discriminated most highly between groups.

**Table 3:** Differences in ITAS scores (items, subscales, and total score) by insulin use

**Item**		**Non-insulin users**		**Insulin users**		***d***
		***M ± SD***	***A/SA%***	***M ± SD***	***A/SA%***	
**1**	Taking insulin means I have failed to manage my diabetes with diet and tablets	3.5 ± 1.3	*58.3%*	2.8 ± 1.3***	*39.4%*	0.54
**2**	Taking insulin means my diabetes has become much worse	4.0 ± 1.0	*80.2%*	3.2 ± 1.2***	*51.0.0%*	0.76
**3^**	Taking insulin helps to prevent complications of diabetes	3.9 ± 1.0	*76.4%*	3.9 ± 1.0	*76.7%*	0.04
**4**	Taking insulin means other people see me as a sicker person	3.3 ± 1.1	*46.3%*	2.7 ± 1.1***	*26.1%*	0.53
**5**	Taking insulin makes life less flexible	3.6 ± 1.1	*58.7%*	2.8 ± 1.1***	*28.9%*	0.71
**6**	I'm afraid of injecting myself with a needle	3.3 ± 1.4	*47.9%*	1.9 ± 1.2***	*10.4%*	1.08
**7**	Taking insulin increases the risk of low blood glucose levels (hypoglycaemia)	3.4 ± 1.0	*46.5%*	3.0 ± 1.1***	*36.1%*	0.45
**8^**	Taking insulin helps to improve my health	3.8 ± .08	*67.7%*	3.9 ± 0.9	*75.5%*	-0.12
**9**	Insulin causes weight gain	3.1 ± .08	*18.2%*	3.5 ± 1.0***	*50.6%*	-0.47
**10**	Managing insulin injections takes a lot of time and energy	3.3 ± 1.0	*40.9%*	2.4 ± 1.1***	*15.3%*	1.04
**11**	Taking insulin means I have to give up activities I enjoy	2.7 ± 1.0	*16.8%*	2.1 ± 1.0***	*6.4%*	0.63
**12**	Taking insulin means my health will deteriorate	2.8 ± 1.0	*18.6%*	2.2 ± 1.0***	*9.2%*	0.57
**13**	Taking insulin is embarrassing	2.7 ± 1.1	*21.6%*	2.2 ± 1.1***	*14.9%*	0.48
**14**	Injecting insulin is painful	3.1 ± 1.0	*32.1%*	2.4 ± 1.1***	*18.9%*	0.67
**15**	It is difficult to inject the right amount of insulin correctly at the right time every day	3.0 ± 0.9	*23.2%*	2.1 ± 1.0***	*12.4%*	0.86
**16**	Taking insulin makes it more difficult to fulfil my responsibilities (at work, at home)	2.8 ± 0.9	*17.8%*	2.0 ± 0.9***	*4.8%*	0.92
**17^**	Taking insulin helps to maintain good control of my blood glucose	3.9 ± 0.8	*74.7%%*	3.9 ± 0.9	*78.7%*	-0.05
**18**	Being on insulin causes family and friends to be more concerned about me	3.6 ± 0.9	*57.7%*	3.0 ± 1.0***	*33.7%*	0.65
**19^**	Taking insulin helps to improve my energy levels	3.3 ± 0.7	*30.9%*	3.1 ± 0.9**	*30.5%*	0.28
**20**	Taking insulin makes me more dependent on my doctor	3.4 ± 0.9	*47.3%*	2.9 ± 1.1***	*33.7%*	0.54
**Total Score**		60.7 ± 10.1		50.2 ± 10.3***		1.03
**Positive Subscale**		14.9 ± 2.4		14.8 ± 2.6		0.04
**Negative Subscale**		51.6 ± 10.2		41.2 ± 9.6***		1.04

M: mean; SD: standard deviation; A/SA: Agree/Strongly Agree; ^ positive ITAS items, **p < .01, ***p < .001.

Scoring: 1 = Strongly Disagree, 5 = Strongly Agree.

There were significant differences between treatment groups for the negative subscale (t (746) = 13.44, p < 0.001, *d* = 1.04) and total ITAS score (t (746) = 13.05, p < 0.001, *d* = 1.03). The percentage of participants who agreed with the benefits of insulin was high, regardless of treatment type, and the mean positive subscale score was not significantly different between groups (t (746) = 0.55, p = 0.582, *d* = 0.04).

Associations between ITAS scores and demographics were comparable between groups. Total ITAS scores were weakly positively correlated with age for both insulin users (r = -0.29, p < 0.001) and non-insulin users (r = -0.18, p < 0.05). No significant relationship was apparent between ITAS scores and diabetes duration for either group (both p > 0.607). Between group ITAS scores did not differ by gender (p > 0.558 for both) or whether participants reported being in a relationship (p > 0.118 for both). Both insulin users (t (247) = -2.17, p = 0.03) and non-insulin users (t (494) = -3.65, p < 0.001) who participated in paid work reported significantly higher ITAS scores (more negative) than those not in paid work.

## Discussion

In replication of the ITAS development paper [15], EFA and reliability tests were conducted on the whole sample and similar results were observed. However, contrary to the original recommendation to use a single ‘Total ITAS’ score, we believe the two-factor structure appears to be a better representation of the 20-item scale, with only ‘weight gain’ not loading on either scale.

In exploring the two-factor solution for the insulin using and non-insulin using samples separately, we found variation in some item loadings. For non-insulin using participants, item 7 (‘increases risk of hypoglycaemia’) did not load onto either subscale. This might be due to a lack of knowledge among non-insulin using participants regarding the potential risk of hypoglycaemia caused by insulin use, though almost half of this sample agreed or strongly agreed that this was a risk. In contrast to the whole sample, item 9 (‘weight gain’) loaded onto the negative subscale for non-insulin users and a number of other variables (items 2, 4, 5, 18) had multiple loadings, while all loading strongest on the negative subscale. Only item 18 loaded >0.4 on both the positive and negative subscale. This might be explained by non-insulin using participants feeling that insulin causing “family and friends to be more concerned about me” would in fact be a benefit of insulin use. Indeed, the item wording is not sufficiently negative for the direction to be clear. For insulin using participants, items 1 (‘insulin means my previous self-care has failed’), 2 (‘insulin means my diabetes is worse’) and 9 (‘weight gain’) were just shy of loading onto the negative subscale (if a stringent criterion of >0.4 was applied) but loaded well when a less conservative criterion (>0.3) was adopted. However these items were the three most highly endorsed among insulin using participants, which suggests they should not be removed from the scale. The forced two-factor solution was the best representation of the 20-item questionnaire for both samples.

Reliability of negative and positive subscales was similar between groups, with the high negative subscale alpha suggesting the possibility for scale reduction. A small number of items performed inconsistently between treatment groups. They maybe candidates for removal or it may be that different items are important for different treatment groups. These items (1, 2, 7, and 9), which also had lower item-total correlations and did not load consistently, were often more highly endorsed by participants than those which performed better statistically, suggesting they have strong face validity. It may be that these items are not performing statistically as well because they are conceptually quite independent of other negative aspects of insulin use, while the items that load well and display high item-total correlations frequently refer to similar barriers to insulin (e.g. the burden of insulin injections in terms of its effect on lifestyle and responsibilities, as well the technical/physical aspects of having to inject).

Given the high internal consistency reliability of the 20-item total scale, the ITAS total score has been recommended for use to quantify overall insulin appraisal [12]. However, in both the ITAS development paper and our current study, Cronbach’s alpha for the 20-item scale is lower than for the 16-item negative scale, and the positive items display very low item-total correlations. Unsurprisingly, a forced one-factor EFA reveals that the four positive items do not load on the factor for either treatment group. Given these results, we propose that the positive and negative subscales should not be combined to create a total score.

Consistent with international findings adults with non-insulin-treated T2DM report significantly more negative attitudes towards insulin use than those using insulin, with total ITAS scores differing by approximately one standard deviation [12,14,15]. Non-insulin using participants reported higher (more negative) scores on 15 of the 16 items, with ‘weight gain’ the only item for which insulin using participants reported more negative appraisals. This is also consistent with previous research [12,14]. With the exception of ‘weight gain’, the ranking of negative appraisals was similar between the two treatment groups; suggesting similar prioritisation of concerns about insulin between groups and differing only by intensity of endorsement. Ceiling effects were present for four ITAS items among non-insulin using participants, but not for the insulin using participants. However, as these ceiling effects are moderate (<33%), are in line with the expected scoring direction of non-insulin using participants, and not displayed among insulin using participants, we suggest that they may not be cause for concern necessarily.

While the ‘negative appraisal’ subscale showed strong discriminatory power, the positive subscale did not differ between treatment groups. Similar to previous studies, we found that participants not using insulin commonly endorsed positive statements about insulin [9,10,12,14,15]. This suggests that concerns about insulin initiation, or psychological insulin resistance, may exist independently of the belief that insulin may be beneficial. This may have implications for clinical care. For example, when counselling patients about their diabetes management options, it may be more beneficial to acknowledge, normalise and then minimise their perceived barriers to insulin use rather than emphasising only the actual benefits of insulin use. However, we recommend that the positive items are retained in the scale for further research purposes such as exploring the subscales association with other variables (e.g. self-care behaviours, optimal insulin taking behaviours). For example, previous research has reported optimal medication taking behaviours to be associated with belief in the benefit of the medication [26].

### Strengths and limitations

The strengths of this study include it being the first study to quantitatively explore PIR in an Australian sample using a validated measure. In addition, it is the only study to further evaluate the psychometric performance of the ITAS since its development. The large sample size and the inclusion of both insulin using and non-insulin using participants to enable separate psychometric analyses are also advantages.

The limitations of Diabetes MILES – Australia are discussed in detail elsewhere [17]. Limitations of specific relevance to the current study are the self-report nature of the diabetes diagnosis for 26% of the sample and the proportion of missing data. The ITAS was developed and validated for use among people with T2DM. Given the self-report nature of Diabetes MILES – Australia, it is impossible to tell whether all participants were accurately classified as having type 2 diabetes. However, it is expected that the following safeguards reduced the likelihood of participants being misclassified. The majority (74%) of respondents had received a pre-determined survey booklet type (specific to their diabetes type and treatment) based on their diabetes diagnosis as reported within in the NDSS [17]. These participants had the opportunity to complete the ITAS only if they were registered with the NDSS as having a diagnosis of T2DM; their data were removed from the current analysis if they subsequently reported a diagnosis of type 1 diabetes within the survey booklet. Online participants (26%) received the ITAS for completion only if they self-reported living with T2DM. Thus, any participant who received a type 1 booklet or self-reported online that they had type 1 diabetes would not have had the opportunity to complete the ITAS. Prior literature concludes that self-reported diabetes diagnosis, not type specific, is reasonably accurate when compared to medical data [27,28]. However, to the author’s knowledge, no research has explored the validation of self-reported diabetes type comparing those with type 1 and those with T2DM.

A further limitation of the current analysis was the missing data, which differed by treatment group, suggesting that the scale may be more acceptable or relevant to participants with T2DM using insulin than non-insulin using participants. It is probable that those non-insulin using participants who skipped the scale entirely perceived it to be irrelevant for them, despite instructions asking non-insulin users “to answer each item based on their current knowledge and thoughts about what insulin therapy would be like”. Hence, we advise future ITAS users to consider including instructions that better emphasise that the questionnaire is to be completed by all participants, not just those already using insulin. After excluding participants who skipped the entire questionnaire, almost three quarters of those with missing data skipped just one item and no particular item displayed substantial non-completion. This indicates acceptability of items among the majority of participants.

Finally, the wording of some ITAS items assumes current or prior use of lifestyle modifications or blood glucose lowering tablets. However, it is possible that a proportion of participants with insulin-treated T2DM have not actively managed their diabetes prior to beginning insulin, and therefore, may find some ITAS questions inappropriate. For example: “Taking insulin means I have failed to manage my diabetes with diet and tablets”. As the Diabetes MILES survey did not ask participants to report previous diabetes treatments, we are unable to clarify what proportion, if any, were prescribed insulin immediately after diagnosis of T2DM. This is a potential limitation of the questionnaire and we recommend that future users consider including assessment of prior diabetes management.

## Conclusions

In the present study, the 20-item ITAS total score explained less variance and displays lower internal consistency reliability than the 16-item ‘negative appraisal’ score. We recommend that calculation of the 20-item ITAS total score be avoided in preference for the 16-item ‘negative appraisal’ score, with close attention paid to the relevance and usefulness of the ‘positive appraisal’ subscale in the given population. Our findings support use of the ITAS in both treatment groups. As perceptions of insulin use appear to vary based on expectation versus actual experience, it is unsurprising that certain items performed inconsistently between groups. The ITAS is a relatively brief and easy to complete questionnaire which may be useful clinically to promote discussion with people with T2DM about their concerns regarding insulin use, or to evaluate interventions to reduce PIR.

## Abbreviations

BIT: Barriers to insulin treatment questionnaire; EFA: Exploratory factor analysis; GLP-1: Glucagon-like peptide-1; ITAS: Insulin treatment appraisal scale; NDSS: National diabetes services scheme; PIR: Psychological insulin resistance; T2DM: Type 2 diabetes mellitus.

## Competing interests

JS has received research funding and consultancy fees from Sanofi Diabetes, in addition to support to attend academic meetings from Sanofi Diabetes and Novo Nordisk. FP has acted as an advisory board member and speaker for Novo Nordisk, and as a speaker for Sanofi-Aventis. He has received a grant from Novo Nordisk to support research and he has received funding for travel and accommodation to attend DAWN2 (Diabetes Attitudes Wishes and Needs) International Publication Planning Committee meetings. FP was also a co-author on the ITAS development and validation paper [15].

## Authors’ contributions

JS conceived The Diabetes MILES Study, and together with FP developed The Diabetes MILES Study International Collaborative. JS is principal investigator of Diabetes MILES – Australia. EHT was project manager of Diabetes MILES – Australia throughout 2011. EHT conducted all data cleaning and analyses of the measures reported here and prepared the first draft of the manuscript. JS and FP provided advice throughout the analysis and interpretation of results. All authors commented on the initial draft, prepared revisions, and approved the final manuscript.
